# Community perspectives on HIV, violence and health surveillance in rural South Africa: a participatory pilot study

**DOI:** 10.7189/jogh.06.010406

**Published:** 2016-06

**Authors:** Nitya Hullur, Lucia D’Ambruoso, Kerstin Edin, Ryan G Wagner, Sizzy Ngobeni, Kathleen Kahn, Stephen Tollman, Peter Byass

**Affiliations:** 1 Institute of Applied Health Sciences, University of Aberdeen, Aberdeen, Scotland, UK; 2 Umeå Centre for Global Health Research, Division of Epidemiology and Global Health, Department of Public Health and Clinical Medicine, Umeå University, Umeå, Sweden; 3 MRC/Wits Rural Public Health and Health Transitions Research Unit (Agincourt), School of Public Health, Faculty of Health Sciences, University of the Witwatersrand, Johannesburg, South Africa; 4 INDEPTH – An International Network for the Demographic Evaluation of Populations and Their Health, Accra, Ghana

## Abstract

**Background:**

South Africa faces a complex burden of disease consisting of infectious and non–communicable conditions, injury and interpersonal violence, and maternal and child mortality. Inequalities in income and opportunity push disease burdens towards vulnerable populations, a situation to which the health system struggles to respond. There is an urgent need for health planning to account for the needs of marginalized groups in this context. The study objectives were to develop a process to elicit the perspectives of local communities in the established Agincourt health and socio-demographic surveillance site (HDSS) in rural north–east South Africa on two leading causes of death: HIV/AIDS and violent assault, and on health surveillance as a means to generate information on health in the locality.

**Methods:**

Drawing on community–based participatory research (CBPR) methods, three village–based groups of eight participants were convened, with whom a series of discussions were held to identify and define the causes of, treatments for, and problems surrounding, deaths due to HIV/AIDS and violent assault. The surveillance system was also discussed and recommendations generated. The discussion narratives were the main data source, examined using framework analysis.

**Results:**

The groups identified a range of social and health systems issues including risky sexual health behaviors, entrenched traditional practices, alcohol and substance abuse, unstable relationships, and debt as causative. Participants also explained how compromised patient confidentiality in clinics, insensitive staff, and a biased judicial system were problematic for the treatment and reporting of both conditions. Views on health surveillance were positive. Recommendations to strengthen an already well–functioning system related to maintaining confidentiality and sensitivity, and extending ancillary care obligations.

**Conclusion:**

The discussions provided information not available from other sources on the social and health systems processes through which access to good quality health care is constrained in this setting. On this basis, further CBPR in routine HDSS to extend partnerships between researchers, communities and health authorities to connect evidence with the means for action is underway.

South Africa is in a state of a health transition, facing a burden of infectious diseases, characterised by high levels of HIV/AIDS and tuberculosis, emerging epidemics of non–communicable diseases (NCDs) including mental illness, extremely high rates of mortality owing to violence and injuries, as well as existing burdens of maternal, neonatal and child deaths. There are considerable pressures on the health system to deal with a complex and dynamic quadruple burden of disease and mortality [1–4]. This paper reports on a study concerned with two leading causes of death in the country: HIV/AIDS and violent assault.

HIV/AIDS has characterized South Africa’s health profile for four decades. Despite constituting 0.7% of the world population, South Africa accounts for 17% of the global burden [5], with an estimated 6.4 million people infected [6]. The distribution of the burden is highly unequal. Prevalence rates in black populations are 40–50 times that of whites, 18–20 times that of Indians and Asians and five–to–six times that of coloreds [6,7]. And in adolescents, risks are eight times higher in females vs males [6]. A host of additional social and structural drivers–mobile populations, over–crowded settlements and exploitative migrant labor–contribute to HIV/AIDS remaining a critical public health challenge [8].

Following an initial period of denial by the Government over the epidemic in the early 2000s, access to antiretroviral therapy (ART) has expanded dramatically through donor initiatives and progressive health policies [9,10]. Currently South Africa operates the world’s largest ART initiative, covering 1.8 million people [11]. Despite achievements, pronounced disconnects between policies and implementation, resulting from ineffective leadership, lack of accountability and inadequate financing, have hampered impacts [12].

Violence is a major cause of death in South Africa [13,14]. The murder of Reeva Steenkamp by the Paralympic champion Oscar Pistorius in 2013 and the Marikana miners’ massacre in 2012 reflect the normalcy of violence at all levels of South African society. At population level, excess homicide is observed according to residence, age, sex and socio–economic status [15]. Studies also suggest that known community members commit almost half (44%) of violent assaults out of jealousy and anger, and often aggravated by alcohol and substance abuse [16,17]. Entrenched inequalities in income and opportunity clearly push disease burdens towards vulnerable populations in South Africa, a situation to which the health system struggles to respond.

Since the first democratic elections in 1994, South Africa has made radical health reforms: a constitutional commitment to the right to health and with stated aims for equity through universal health coverage [18,19]. Progress towards the Millennium Development Goals (MDGs) has been attributed to the expansion of primary care and free health services for expectant mothers and children under five years [18], and a national health insurance system is currently being implemented [11]. In spite of these achievements however, the health system is deeply unequal. Colonial and apartheid legacies persist in unaccountable governmental systems, inadequate stewardship and financing, inefficient management and insufficient resources [20]. Most recently, neoliberal macroeconomic structural adjustment policies that prioritize growth over redistribution have deepened the divide between public and private care [12,21].

In a context of complex disease burdens, multiple and intersectional health inequities and weak health systems, civil registration and vital statistics (CRVS) play a critical role [22]. Routine health information that is reliable and robust is a critical means to strategize, evaluate and monitor progress [23] as well as foster security and citizenship more broadly [24]. The issue requires special attention following estimates that over two–thirds of deaths worldwide pass without registration [25], with over three quarters of these belonging to regions in sub–Saharan Africa and South–East Asia [26]. Although South Africa has a vital registration system for births, deaths and medical cause of death that is comprehensive in relation to other countries in the region [27], the system does not allow for correction of misclassifications in death certificates and audits have identified errors in up to 94% of records on HIV/AIDS deaths [28].

There is an urgent need for the health of marginalized groups to be accurately represented in this context. The objectives were therefore to develop a method to elicit perspectives of local communities in an established health and demographic surveillance site (HDSS) in rural north–east South Africa on two leading causes of death: HIV/AIDS and violent assault, and on HDSS as the means through which health information is generated in the locality. The broader aim was to demonstrate the utility of routinely consulting communities in HDSS.

## METHODS

### Study setting

**Figure 1:**
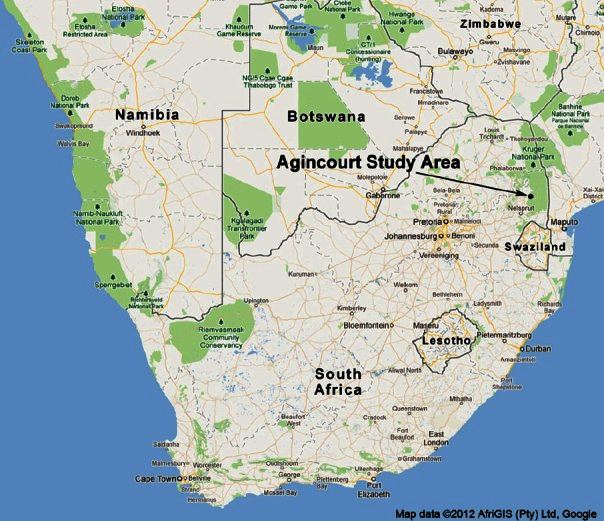
Agincourt Health and Socio-Demographic Surveillance Site (HDSS) in Bushbuckridge Municipality, Mpumalanga Province, South Africa.

The study setting was the Medical Research Council (MRC)/Wits Rural Public Health and Health Transitions Research Unit in rural northeastern South Africa, which oversees the Agincourt Heath and Socio–Demographic Surveillance Site (HDSS). The Agincourt site was established in 1992 in response to an absence of vital information on rural populations in South Africa, and has conducted annual censuses since collecting information on vital events (births, deaths bind migrations) in a population of approximately 110 000 occupying 21 000 households across 31 villages (**Figure 1**) [29,30]. Agincourt established the Learning, Information, dissemination and Networking with Communities (LINC) group in 2004 to enable community participation in research and governance. LINC enhances research quality through the feedback of research results to community stakeholders [31]. Through these activities, the Agincourt HDSS tracks population health over time, measures the impact of interventions, supports community research and addresses gaps in population health data [32].

### Participatory approach

The research adopted a community–based participatory research (CBPR) approach [33,34]. This was based on the premises that deaths identified through routine health surveillance have social and health systems determinants, and the mechanisms through which these factors influence health outcomes can be reliably identified with local knowledge [35]. Given the time and resources available, communities participated in identifying and defining health problems only. Other than in terms of health surveillance, the communities did not formulate remedial actions, and these were not implemented or evaluated. The research was of a pilot nature exploring feasibility and providing formative data as a basis form which to develop fuller forms of participation in the study setting.

### Participant recruitment

Three villages were selected in the surveillance area in which to convene the discussion groups on the bases of demographic variation (**Table 1**) and feasibility within the time and resources available. LINC staff then approached women of reproductive age (WRA), family members, traditional healers, religious leaders, community health volunteers, health workers, village officials, and community leaders in villages to convene discussion groups that broadly represented the community. To mitigate against any potential bias as for result of social and power differentials in the groups, the group consisted of women only in one village (**Table 2**).

**Table 1:** Characteristics of selected villages*

		Village	
	**A**	**B**	**C**
Number of households	1178	932	647
Population, total	6158	4827	3705
Population, male	3005	2305	1781
Population, female	3147	2522	1924
Population, children under 5	647	513	458
Population, children of school age	1911	1410	1167

*Source: Household data collected by the MRC/Wits Rural Public Health and Health Transitions Research Unit (Agincourt), June 2013 [36].

**Table 2:** Composition of discussion groups

	Discussion group		
Participants*	A	B	C†
Women of reproductive age	1	1	2
Family members‡	2	2	2
Traditional healers	1	1	2
Religious leaders / elders	1	1	2
Community health volunteers§	1	1	
Village officials§	1	1	
Community health providers	1	1	
TOTAL	8	8	8
			24

*All participants recruited were 18 y or older. Although participants typically had >1 role in the community, one primary role per individual was adopted for the purposes of convening the focus groups. Primary roles were also confirmed with participants.It was acknowledged that people with working arrangements, particularly village health workers and village officials’ availability for five consecutive weekly meetings could be compromised. We also acknowledged the ethical imperative of engaging participants who would otherwise be involved in earning income and/or in the provision of public services. Participant recruitment was based on the compositions above with a degree of pragmatism and flexibility towards those committing to the process, and with careful consideration of minimising disruption to local public services.

† Group C was a womenonly group to mitigate against the power differentials arising from the heterogeneous constituency of the groups.

‡Close relative: parents, grandparents, siblings, children, in–laws, nieces, nephews and cousins.

§Group C was a women only group to mitigate against the power differentials arising from the heterogeneous constituency of the groups.

An introductory meeting was held where the purpose, planned activities and outputs of the study were described. Those willing to participate were enrolled, written consent was taken and written information on the study was provided. A total of 24 participants were recruited into three village–based groups of eight participants, which operated independently in a series of weekly discussions on four selected health conditions, plus the introductory meeting described above (**Table 3**). The conditions were selected on the bases of high prevalence (HIV prevalence is 45% among men and 46% among women aged 35–39 in the Agincourt HDSS [37] and mortality from violent deaths is “outstandingly high” [38]) and in terms of socio–cultural relevance [39–42].

**Table 3:** Schedule of focus group discussions

Week/topic	1	2	3	4	5	
Focus Group	Recruitment/Introduction	Stroke	HIV/AIDS	Violent assault	Epilepsy and feedback	Total meetings, per group
A	A, 1	A, 2	A, 3	A, 4	B, 5	5
B	B, 1	B, 2	B, 3	B, 4	B, 5	5
C	C, 1	C, 2	C, 3	C, 4	C, 5	5
Total number of focus group discussions						15

### Data collection

A qualitative approach to data collection and analysis was adopted to elicit the collective perspectives of the village–based groups on the relationships between medical problems and their social and health systems determinants. The focus group discussion (FGD) method was used to encourage participation, to capitalize on communication between participants and to explore people’s knowledge to gain an understanding of the collective norms and attitudes surrounding the two conditions [43,44]. A series of five weekly FGDs of 1.5–2 hours were held in each of the three villages, 15 FGDs were held in total.

A senior qualitative investigator (SN) with detailed knowledge of the area facilitated the discussions. SN presented data gained via the annual census on the conditions to the groups and facilitated discussions on this basis. Topic guides were prepared for the meetings in which the conditions, their causes, treatments, and the means through which information on them was generated in the locality (ie, HDSS) were discussed. The discussions were audio recorded and translated from the local language xi–Tsonga into English and transcribed. Two investigators took observational field notes and provided generally assistance during the meetings (LD and KE).

### Data analysis

The narratives and field notes were the main data sources. Towards the end of the data collection, the groups were presented with and discussed a preliminary analysis to determine the plausibility and relevance of early interpretations of the discussions (**Panel 1**). Following completion of the data collection, a detailed analysis of the discussion transcripts was undertaken using framework analysis (NH). Framework analysis is a flexible tool to analyze qualitative data with the aim of creating a descriptive overview of an entire data set [45]. This method involved familiarization and coding of the data followed by preparation of summaries/charts to map the range of views on the phenomena of interest [46]. The steps of the framework analysis are summarized in **Table 4**. NVivo software (QSR International, London, UK) was used for data management and coding.

**Table 4:** An adapted framework analysis approach [45,46]

Stage	Description
1. Immersion and organisation	An initial organisation of data according to pre–determined (deductive) categories, as well as to preliminary emergent (inductive) themes.
2. Development of coding frameworks	The development of thematic, or coding frameworks that resulted from Stage 1.
3. Application of coding frameworks	The thematic frameworks are applied to the data to code or index it. This is done iteratively, until no new themes emerge (“thematic saturation”).
4. Preparation of thematic summary grids	Thematic summaries prepared: grids of dominant and recurrent themes prepared with related themes and sub–themes in columns and respondents (or groups of respondents) as rows. This allows large volumes of narrative data to be distilled, and allows for the identification of patterns within and between narratives.
5. Interpretation	Establishing associations between themes to construct descriptive and explanatory accounts of the phenomena of interest.

### Ethical considerations

Ethical considerations related to the research process and outcomes were integrated into the study design [47]. All participants gave informed consent that guaranteed anonymization of all identifiable data in study reports, and assured participants that they were free to leave the study at any time and for any reason. The study protocols were peer reviewed to determine local, methodological and substantive relevance. The Human Research Ethics Committee at the University of the Witwatersrand (clearance #M121039) and the Mpumalanga Province health authority also reviewed and approved the study protocol.

## RESULTS

This section presents the results of the analysis illustrated with verbatim quotes from the transcripts of the FGDs.

### Causes of HIV/AIDS related mortality

Participants reported that widespread financial insecurity, often coupled with material desires, encourages young people to trade sex for immediate financial gain, often at the expense of their long–term health. The participants also described how gendered roles and expectations in relationships, combined with the need to maintain social and financial security, constrain safe sex practices. The introduction of condoms was specifically reported to induce doubts among men about the fidelity of their partners, which threatened the relationships that are sources of social and financial support for many women.

“Poverty is one of the main issues when it comes to this disease. People still take risks because they want money to make a living.” [Group A; Village Official]

“…Most men … don’t believe that they should use a condom with a woman that they have a baby with or a wife.” [Group A; Village Official]

Traditional practices were also reported as causative. A practice called *Milo* was noted in this regard. *Milo* is a traditional cleansing ceremony performed following the death of a husband. The ceremony involves widows having unprotected sex with a mentally ill man for 7–14 days to prepare for future sexual relationships. Due to the presence of few such individuals in communities, the same people were rotated among widows in villages for *Milo*. Due to the likelihood that women were widowed as a result of HIV/AIDS, *Milo* was identified as a route of transmission in communities.

“…they don’t know the health status of [persons involved in the ceremony] and it might happen that he infect[s] my mother … because he does it [with] several people who needs the ceremony.” [Group A; WRA]

### Treatments for HIV/AIDS related mortality

The use of traditional medicine for HIV/AIDS was described in detail. Traditional healers were often portrayed negatively in these discussions, as money–minded and misguiding. Participants reported instances in which healers, after identifying symptoms of HIV/AIDS, advised patients to undertake training to become a traditional healer themselves. Participants expressed suspicions that this was a strategy to gain revenue, and one that discouraged individuals from seeking and receiving medical treatment.

“…They were taken to the traditional healers and some …say it’s a call from the ancestors to become a *Sangoma* [traditional healer]. She will go there and start to be a healer but at the end the traditional healer will chase her when he saw that she is not getting better (participants laugh).” [Group B; WRA]

Participants also reported that HIV patients would often be diagnosed and treated for *Tindzaka*, a disease believed to develop due to unprotected sex (as with HIV/AIDS) when mourning a death. Participants described treatments for *Tindzaka* involving cutting the skin of the patient, putting a mixture of herbs in the cut, and making the patient inhale the smoke of the burnt herbs. The participants also described medical treatments and self–help actions known to help patients to cope with, and prevent worsening of, the condition. The latter included acceptance of the situation, familial support, positive changes in diet and lifestyle, reductions in alcohol consumption, and a complete stop on sexual activity.

“…As a family we need to give moral support to the person and show love to him, ensure that he eats and drinks the medication.” [Group A; Village Official]

In terms of medical treatments, participants reported that ART had proved beneficial for the health of people living with HIV/AIDS. Many people however were reported to experience pain, nightmares and excessive hunger with ARTs, which constrained compliance in some cases.

“... ARTs are very painful when you are using them. That is why some are defaulting.” [Group B; Village Official]

**Figure 2:**
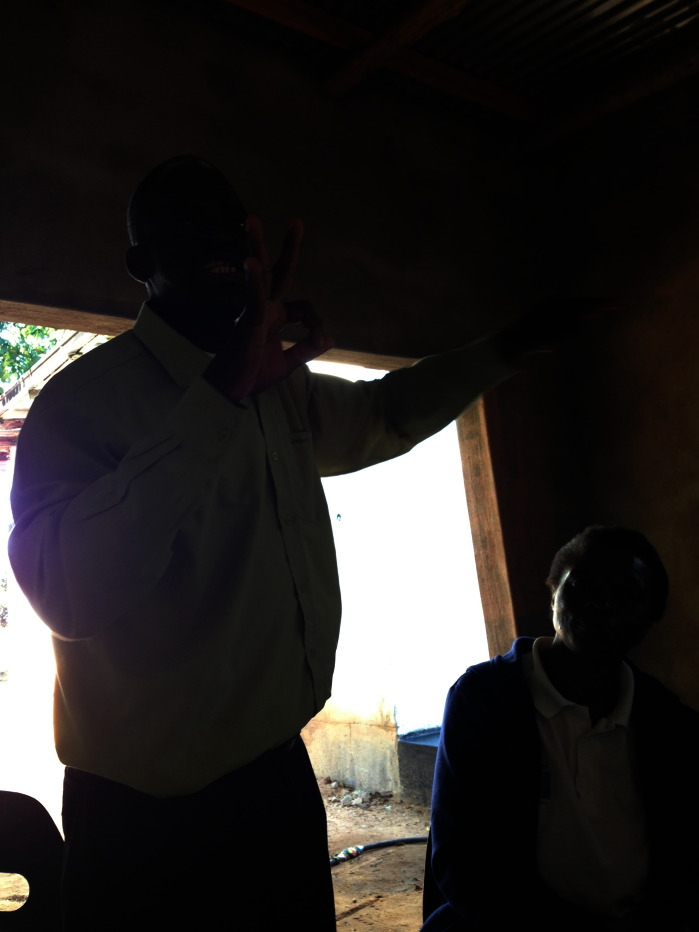
Focus group discussion (FGD) participant showing hand gestures used by medical staff to disclose HIV/AIDS status. Permissions were secured from participants for the reproduction of this image.

Problems with patient confidentiality were reported in all groups as a major issue with medical treatment for HIV/AIDS. Although some clinics counselled patients before disclosing test results, elsewhere providers reportedly disclosed patients’ status using non–verbal gestures (**Figure 2**). Casual and stigmatising attitudes among nurses when dealing with the personal details of patients were also described. Participants appeared uninformed of the pathways through which disciplinary actions for such breaches of confidentiality could be initiated.

“…HIV/AIDS [is] associated with prostitution, then when at the clinic say those with files [indicating those who are HIV/AIDS positive] come to this side then we look at them and say these are the ones who are not behaving well.” [Group B; Village Official]

“…our people don’t know about the channels to be used when they want to lay a complaint.” [Group A; Village Official]

The insensitive handling of patient information had pronounced consequences reported to create fear and discomfort among individuals and often resulted in delayed or no testing. Individuals in communities reportedly sought care long distances from their local facilities to avoid regular breaches in patient confidentiality. Delayed care and transportation costs were recounted as key factors in care seeking decisions in these circumstances.

“… Many people are dying in their houses without consulting the clinic because they don’t have faith in the clinic.” [Group A; Traditional Healer]

Participants also described how fears over loss of financial security, social and emotional support delayed people seeking testing and treatment. The reported behaviours of providers and patients underscored pronounced stigmatisation of people living with HIV/AIDS.

“... if he can find that he is HIV/AIDS he will think that he is dead, [participants laugh] he will live in fear you see.” [Group B; Village Official]

The participants also described unintended consequences of social welfare schemes that make support payments to patients with CD4 counts below a certain threshold. Participants described how patients deliberately discontinue ART to continue to qualify for the welfare grant to safeguard a predictable income, but at the expense of continuing treatment.

“…Some when they realize that his CD4 count is getting better and he realize that they are going to take away the grant and he will struggle … he stops taking his treatment and he will remain sick.” [Group B; Village Official]

### Causes of mortality owing to violent assault

Poverty was also a recurring theme in the discussions on the causes of violent deaths. Participants explained how situations of poverty force individuals to take loans and incur debt and how the needs for financial and social security encourage women to have multiple partners. Non–repayment of credit and disloyalty in relationships were described to give rise to violent conflict with fatal consequences.

“… some people have a lot of credit and… he is not paying, like the loan sharks if you don’t pay them they can beat you until you die.” [Group A; WRA]

Participants also recounted excessive drinking and drug abuse as precursors to fatal assaults. These were reported to involve sexual assaults, robberies and criminal gangs resulting in general increase in violence in the community. The narratives also revealed how conflict between couples can result from wives publicly disrespecting their husbands. Alcohol and drug abuse were again reported to exacerbate conflicts.

“… drunk people will fight for something minor; they will even fight just because the other person stepped on his toe.” [Group B; Village Official]

The women–only group portrayed men as the cause of familial violence. Specific issues reported in this group were the discovery of dishonesty and affairs. The heterogeneous groups, by contrast, reflected on the social acceptability of male violence as a means by which men establish control and dominance within the household and wider community.

“… according to our tradition, a women is always a women and the man has the upper hand at home.” [Group A; Village Official]

Conflicts over property ownership, asset distribution, food, money, lack of discipline and guidance between siblings who had lost parents were also reported. Additionally, favoritism of siblings by parents who were present was also reported as a source of tension, especially in second marriages.

### Treatments for mortality owing to violence

The groups described several traditional and religious practices employed to reduce conflict and violence in families. These included husbands drinking traditional herbs to induce calm. In one village, an instance where the pastor took the name of the Lord, holding the t–shirt of the violent husband to reduce his anger was recounted. The participants expressed confidence in the effectiveness of traditional and religious practices.

Non–disclosure of violence due to stigma and the need to maintain socio–economic security were also recounted. Many participants reported how assaults are hidden from the police with women using home–based therapies to treat wounds so as not to compromise household stability and income. These actions protected violent spouses, again often with fatal consequences.

“…They are scared that they will arrest their husband because they are the ones providing food on the table for the family. That is why some they keep it to themselves until they are beaten to death.” [Village B; Village Official]

Views on hospital treatment for assault were generally positive. Participants reported that hospitals provided timely care and engaged with the authorities to report crimes and protect victims. Despite this however, poor quality services in public health facilities were also described. Participants reported insensitive behaviours of nurses, unskilled medical staff and rigid shifts often leading to long waiting hours, sometimes with fatal outcomes.

“… hospital staff will even put a policeman to guard him, this shows that they not only want to heal people but also want the law to take its course.” [Group B; Pastor]

“… nurses at the hospital are very cruel and rude… they never cared, they said I must pick the person and lay him in bed and the person died.” [Group C; Community Health Provider]

Ambulances were frequently reported as lacking, as were the high transportation costs and poor access to emergency services. Unwillingness to take injured persons to hospitals in private vehicles was expressed in some villages. The participants also reported that at the police station, men who report their wives face disrespectful behaviour from officers.

“…many people are scared of blood … when you call people with cars when they see that the person is heavily bleeding they won’t take that person to hospital because they think he might die in their car.” [Group C; Traditional Healer]

“…when they arrest a man they will always come to his house while he is in jail to get a police statement and they use that as an excuse to get your wife while you are away [participants laugh].” [Group B; Village Official]

### Health surveillance

The discussions also sought views on the procedures and outcomes of longitudinal health surveillance in the communities. The participants acknowledged the benefits of health surveillance in terms of understanding and awareness of health issues in communities.

“Health surveillance staff can help us in understanding more about HIV/AIDS issues and also help those who are left behind in being able to understand more about the disease and if needs be they take the treatment in a correct manner.” [Group A; Community Health Provider]

The participants developed recommendations for the surveillance system. These included educational, financial and employment support to families whose needs were identified through routine surveillance. Other suggestions referred to HDSS partnering with community social workers to provide guidance and improve awareness on health and disease in the community.

“…I think if you investigate the cause of death it will be much better if you can come back and offer assistance to the family in a sense of checking their health status.” [Group C; WRA]

Participants also stated that field–workers should show sensitivity and patience during household surveys. Additionally, participants reported higher levels of acceptability with field–workers who were not known in their community. As well as for impartiality and confidentiality, this was reported to improve the validity of information provided.

“... you are from [an]other village and come to investigate about death I will tell you the truth because I don’t know you. It will be between you and me.” [Group B; Family Member]

The participants stated that the feeding back results of routine surveillance should incorporate an individual approach to personally inform families of the outcomes of cause of death conclusions gained through elements such as Verbal Autopsy (VA). Participants also stated that one–to–one feedback should maintain strictly confidentiality, protecting families from potentially harmful consequences related to the disclosure of stigmatised conditions.

“…if you tell the whole community that so [individual] was killed by so [cause of death] it will be a disaster.” [Group A; Pastor]

## DISCUSSION

The community views on HIV/AIDS and violence were broadly consistent and common issues were identified across the groups. The discussions on HIV/AIDS revealed serious problems with respectful care, confidentiality and patient dignity, while the discussions on violence reflected a patriarchal society with pervasive use of violence as a means of establishing control, social power and position within households and the wider society. The discussions on both conditions revealed the extent to which economic and social insecurities and traditional beliefs influence health and health behaviours. Specific issues included: norms of unsafe sex, widespread prostitution, debt, acceptance of domestic violence, and stigma around disclosure.

These issues did not exist in isolation. The discussions revealed how, in a context of pervasive vulnerabilities, actions to maintain social support and position in the immediate term (eg, by not practising safe sex or not seeking of HIV testing or treatment) were often necessary to prioritise over actions to safeguard health in the longer term. These actions and norms were further reinforced by the lack of effective health system responses such as the denial of confidentiality of health status and lack of emergency transport. The results can therefore be considered in terms of convergent forms of disadvantage and exclusion that exert strong influences over people’s ability to protect their health.

More generally, poverty was repeatedly reported as a root cause for the emergence, transmission and exponentiation of mortality owing to HIV/AIDS and violent assault. Literature on the social diagnosis approach [48,49] and fundamental cause approach [50] assert that poorer households face problems in the availability, accessibility, acceptability and affordability of health care. These issues were clearly observed in the problems reported including unavailable emergency transportation, far–away clinics, expensive transport rental services, poor quality of care reflected through long waiting hours, limited hospital resources and staff, and poor confidentiality and insensitive behaviours on the part of health providers.

The South African health system is deeply divided as a result of historical colonialism, apartheid and, more recently, macroeconomic policies imposing neoliberal structural adjustment [51,52]. A recent assessment of equity in the health system in South Africa concludes that despite progressive financing, the distribution of health benefits remains distinctly pro–rich [53]. Entrenched poverty and social inequality, divisions between public and private care, and disconnects between policy and ineffective implementation, have deteriorated public sector personnel and facilities. The implications of poverty as a root cause of mortality in an unequal health system, in which deep social norms of eligibility for care linked to ability to pay, are important and should be a focus of future research.

The discussions provided information not available from other sources on the social and health systems mechanisms through which access to good quality health care is constrained in this setting. The routine engagement of marginalised group in the development of health information, coupled with HDSS for measuring and attributing progress to interventions developed and implemented is a clear avenue for further research [54]. It is encouraging that South Africa has a constitutional commitment to the right to health that centralises community participation in primary health care [55] that has been institutionalised, albeit with variable success, in Community Health Committees in the Western Cape [55]. Further CBPR in the Agincourt HDSS is currently underway to extend the partnerships initiated in this study between communities, researchers and health authorities. The intention is to develop co-constructed practical knowledge built from multiple perspectives, which can be readily embedded in local policy context [56,57].

The community views on health surveillance were largely positive reflecting established public engagement in Agincourt. Suggestions for modifications to an already well–functioning system related to ensuring that surveillance is respectful of loss, grieving and mourning, and for confidentiality and sensitivity when discussing deaths of relatives at the individual level. Extending ancillary care obligations with the provision of support to families in situations of bereavement and impoverishment were also suggested. A recent study on the cultural acceptability of health surveillance supports this finding, recommending that HDSS sites prioritize community sentiments and traditions in data collection and dissemination [58]. Further work on the balance between collective utilities and the protection of individuals in routine surveillance will further strengthen the activities [59,60]

### Strengths and limitations

The degree to which participatory principles were adopted in the study was limited. Given the time and resources available, communities participated in identifying and defining health problems only. Research questions and study designs were largely determined prior to contact with communities, as were the conditions that were discussed. Other than identifying modifications to the health surveillance system, the groups did not discuss remedial actions for services and neither implemented nor evaluated strategies to respond to the issues identified.

Despite limitations in the nature and extent of participation, the study demonstrated that consulting communities offers unique perspectives on the social and health systems components of mortality. Acknowledging principles of CBPR related to developing sustained and authentic partnerships and mutual agendas between communities and researchers [61], the study has served as a basis upon which to design and implement a larger participatory action research (PAR) process in Agincourt. This work will develop a methodology suitable for application in other locations that promotes empowerment and social inclusion in health systems, with capacity building and evidence–based advocacy [62].

Otherwise, the study was conducted in a defined area using qualitative methods and the findings may not necessarily be relevant to different contexts and settings. It is maintained however that qualitative enquiry seeks to provide authentic representations rather than generalizable findings [63,64], and that in terms of the process, CBPR is concerned with changing academic research paradigms through more inclusive collaboration [61], and building partnerships though formative, feasibility and pilot data [65], to which progress has been achieved.

The study also explored perceptions on sensitive issues that were discussed in groups. This may have been subject to limited disclosure and so risk of bias. The differences in perspectives between the discussion groups convened to represent the community vs those that consisted of women only were noteworthy however, and suggest that the engagements may have been to a sufficient degree, authentic and representative of collective viewpoints.

**Figure 3:**
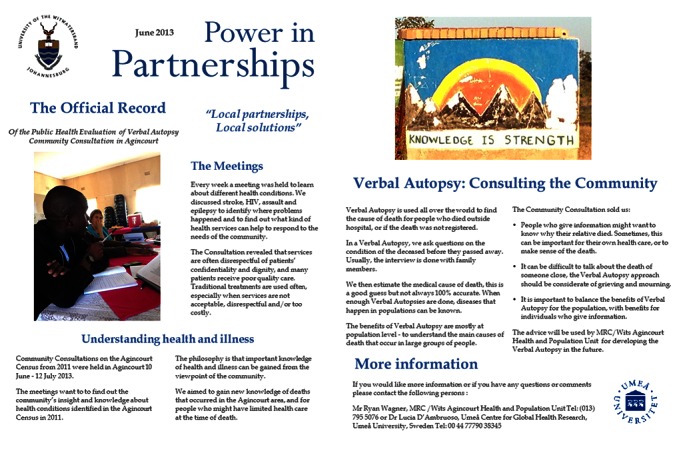
Preliminary analysis presented to discussion groups in the final meeting.

The combined inductive/deductive approach to data collection and allowed flexibility to reveal unanticipated aspects of the community’s perspectives on HIV/AIDS, violence and health surveillance, as well as considering issues identified a priori. A notable result in this sense relates to the unanticipated consequences of the welfare grant awarded on the basis of CD4 counts and how this acts as a disincentive to treatment compliance. Finally, the presentation and discussion/confirmation of the preliminary analysis served as a validity/integrity check with the participants to ensure rigor while reflecting on the phenomena of interest (**Figure 3**).

## CONCLUSIONS

Eliciting community views on the long–standing challenges of HIV and violence in South Africa provided information not available from other sources on the mechanisms through which social, economic and health systems factors influence the accessibility and acceptability of heath care. The discussions also revealed how these factors combine and converge to seriously and negatively constrain the extent to which people can engage in behaviours that safeguard long–term health. Health planning must take account of the social aspects of mortality in service organisation and delivery in future. The results also indicate the need to address pervasive disenfranchisement of rural and poor communities.

In the context of HDSS, systematic documentation of population health and demographic data coupled with validation and co–production of health knowledge in a process connected to the health system at different levels may provide a means to improve evidence–based public health care services and address existing knowledge gaps. As stated by Scott–Samuel on Health Impact Assessments (HIA): “The identification and production of evidence that includes the interests of less powerful groups is a priority for HIA and would be furthered if a human rights–based method of HIA were developed.” [66]. Further participatory work in the Agincourt HDSS is underway to explore the potential to enhance survey data, and to provide a basis from which to develop partnerships between researchers, communities and health authorities in order to connect robust evidence with the means for remedial action.
